# Impact of maternal mental health interventions on child-related outcomes in low- and middle-income countries: a systematic review and meta-analysis

**DOI:** 10.1017/S2045796020000864

**Published:** 2020-10-19

**Authors:** W. A. Tol, M. C. Greene, M. E. Lasater, K. Le Roch, C. Bizouerne, M. Purgato, M. Tomlinson, C. Barbui

**Affiliations:** 1 Section of Global Health, Department of Public Health, University of Copenhagen, Copenhagen, Denmark; 2 Department of Mental Health, Johns Hopkins Bloomberg School of Public Health, Baltimore, MD, USA; 3 Program on Forced Migration and Health, Columbia University Mailman School of Public Health, New York, NY, USA; 4 Mental Health and Care Practices, Gender and Protection, Action contre la Faim, Paris, France; 5 WHO Collaborating Center for Research and Training in Mental Health and Service Evaluation, Department of Neuroscience, Biomedicine and Movement Sciences, University of Verona, Verona, Italy; 6 Institute for Life Course Health Research, Department of Global Health, Stellenbosch University, Cape Town, South Africa; 7 School of Nursing and Midwifery, Queens University, Belfast, UK

**Keywords:** child health, low- and middle-income countries, maternal mental health, perinatal

## Abstract

**Aims:**

Observational studies have shown a relationship between maternal mental health (MMH) and child development, but few studies have evaluated whether MMH interventions improve child-related outcomes, particularly in low- and middle-income countries. The objective of this review is to synthesise findings on the effectiveness of MMH interventions to improve child-related outcomes in low- and middle-income countries (LMICs).

**Methods:**

We searched for randomised controlled trials conducted in LMICs evaluating interventions with a MMH component and reporting children's outcomes. Meta-analysis was performed on outcomes included in at least two trials.

**Results:**

We identified 21 trials with 28 284 mother–child dyads. Most trials were conducted in middle-income countries, evaluating home visiting interventions delivered by general health workers, starting in the third trimester of pregnancy. Only ten trials described acceptable methods for blinding outcome assessors. Four trials showed high risk of bias in at least two of the seven domains assessed in this review. Narrative synthesis showed promising but inconclusive findings for child-related outcomes. Meta-analysis identified a sizeable impact of interventions on exclusive breastfeeding (risk ratio = 1.39, 95% confidence interval (CI): 1.13–1.71, ten trials, *N* = 4749 mother–child dyads, *I*^2^ = 61%) and a small effect on child height-for-age at 6-months (std. mean difference = 0.13, 95% CI: 0.02–0.24, three trials, *N* = 1388, *I*^2^ = 0%). Meta-analyses did not identify intervention benefits for child cognitive and other growth outcomes; however, few trials measured these outcomes.

**Conclusions:**

These findings support the importance of MMH to improve child-related outcomes in LMICs, particularly exclusive breastfeeding. Given, the small number of trials and methodological limitations, more rigorous trials should be conducted.

## Introduction

Mental health is critical to public health and contributes substantially to the global burden of disease (Whiteford *et al*., 2015). In low- and middle-income countries (LMICs), there are few resources to address this burden, resulting in large numbers of people with mental health concerns not receiving treatment (Demyttenaere *et al*., 2004). Calls have been made to make evidence-based treatments for mental disorders more accessible by integrating them into non-specialised health settings, such as primary, maternal and child care systems (Lancet Global Mental Health Group *et al*., 2007).

There are a number of compelling reasons to integrate mental health services into routine maternal and child health care in LMICs. First, mental disorders in the perinatal period are common and disabling (Baron *et al*., 2016). Second, maternal mental disorders are associated with poor child development and health (Surkan *et al*., 2011). Third, maternal and child health care settings provide good entry points for identification and treatment of maternal mental disorders because of the relatively good uptake of antenatal care in LMICs. Fourth, treatments for maternal mental disorders have been evaluated as effective in multiple LMICs and existing treatment guidelines for non-specialised providers include specific recommendations for pregnant women (Rahman *et al*., 2013).

Despite demonstrated links in the epidemiological literature, few systematic investigations have been conducted to examine whether maternal mental health (MMH) interventions can reduce potential negative impacts on children's outcomes. The aim of this study was to conduct a systematic review and meta-analysis on this topic. Specifically, our research question was: do interventions with a dedicated psychiatric or psychosocial component delivered to pregnant women and mothers during the perinatal period improve children's health and development in LMICs relative to standard antenatal care or interventions lacking a dedicated psychiatric or psychosocial component?

## Methods

### Search strategy and selection criteria

We searched PubMed/MEDLINE, PsycInfo, Cochrane CENTRAL, Embase, Web of Science, CINAHL, Popline, several grey literature sources (Global Health Library, UNFPA, UNICEF, WHO, World Bank, Emergency Nutrition Network, ALNAP and Eldis) and trial registration websites (clinicaltrials.gov). The searches were conducted through May 2020 without date, publication or language restrictions. Search strategies contained terms describing the perinatal period (e.g. ‘prenatal’, ‘postpartum’), mental and psychosocial health (e.g. ‘psychosocial’, ‘anxiety’, ‘depression’), LMICs (e.g. ‘low-income’, ‘developing country’, list of LMICs), randomised trial (e.g. ‘randomized’) and child development (e.g. ‘child growth’, ‘child development’, ‘nutrition’; online Supplementary material).

Randomised controlled trials (RCTs) were eligible for our systematic review if the study: (1) described interventions delivered during the perinatal period, defined as pregnancy through 1-year post-partum; (2) incorporated an MMH intervention component; (3) included a MMH outcome; (4) was conducted in an LMIC (http://data.worldbank.org/about/country-and-lending-groups) and (5) included a child health, nutrition or development outcome. We retained the child outcomes for inclusion broad since this is (to our knowledge) the first systematic review and meta-analysis on this topic. All non-randomised, non-controlled studies were excluded. We did not limit our results to studies that restricted their samples to women with mental health problems.

Two independent reviewers assessed titles and abstracts from all searches. English and Spanish full texts were retrieved for potentially relevant articles and assessed by two reviewers independently to evaluate eligibility. Inter-rater reliability in the full text review was 74.4%. Articles and abstracts in other languages (two in Farsi) were assessed by a single reviewer that was fluent in the language. This reviewer worked with another member of the research team to review eligibility criteria. Discrepancies were resolved through discussion or consultation with a third reviewer.

### Data collection, risk of bias assessment and GRADE certainty of evidence

Two reviewers independently extracted data on study design, sample, study conditions, child-related outcomes, results and risk of bias for each included trial (MCG, MEL, see ‘Acknowledgements’). Quantitative results were extracted using the unadjusted means and standard deviations for continuous outcomes and the number of events and denominator for dichotomous outcomes. The risk of bias assessment followed the Cochrane Risk of Bias tool where reviewers rated several potential sources of bias as ‘high’, ‘low’ or ‘unclear’ risk in relation to random sequence generation, allocation concealment, masking of participants/personnel, masking of outcome assessors, attrition, reporting and any other sources of bias of each trial (Higgins and Greene, 2011). We considered overall risk of bias to be high if trials displayed high risk of bias in two or more of these seven domains. Discrepancies were resolved through discussion.

We employed the GRADE approach to assess the overall certainty of evidence and to interpret findings (Barbui *et al*., 2010). We adhered to the standard methods for the preparation and presentation of results outlined in the Cochrane Handbook for Systematic Reviews of Interventions and PRISMA guidelines (Higgins and Greene, 2011). We included the following outcomes in the GRADE evidence profiles: exclusive breastfeeding, cognitive development, psychomotor development, low birth weight, weight (continuous), height (continuous), underweight (i.e. weight-for-age *z*-score <−2), stunting (i.e. height-for-age *z*-score <−2) and weight-for-height.

### Data analysis

Narrative synthesis: included trials were compared with respect to population, intervention, measurement and methodological features that may contribute to clinically relevant heterogeneity in the synthesis of the results. Reporting of these results followed PRISMA recommendations.

Quantitative synthesis: data from included trials were pooled using a random effects model for outcomes reported in at least two trials and expressed as relative risk (RR) for categorical data, and standardised mean difference (SMD) for continuous data. For categorical outcomes with evidence supporting an intervention effect across more than one study, we calculated the number needed to treat (NNT) to provide benefit (Furukawa *et al*., 2002). Review Manager was used for all analyses (The Nordic Cochrane Center, 2014). Data from cluster RCTs were adjusted with an intracluster correlation coefficient (ICC). If the ICC was not available, we assumed it to be 0.05 (Higgins and Greene, 2011). Below, we report intention-to-treat analyses including all randomised patients.

We conducted a sub-group analysis by intervention type: (1) focused MMH interventions (i.e. interventions mainly aimed at improving MMH) and (2) integrated interventions (i.e. interventions that included a mental health focused component, but also focused on other outcomes). We evaluated publication bias for outcomes that included more than ten studies.

## Results

**Fig. 1:**
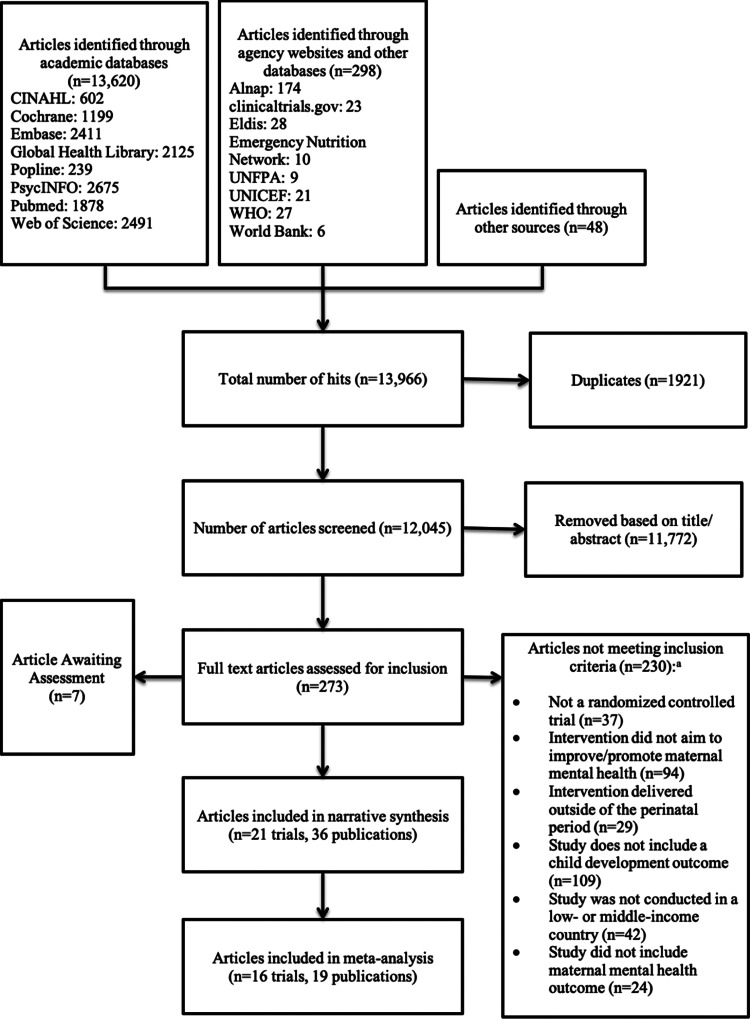
**.** PRISMA flow chart summarising selection of included studies.

Searches yielded 13 918 results, with an additional 48 records identified through cross-referencing and expert recommendation (Fig. 1). After removal of duplicates (*n* = 1921), 12 045 articles were screened. Reviewers identified 273 articles that were potentially relevant and thus included in full text screening. Thirty-six articles representing 21 randomised trials met criteria for inclusion in this systematic review and seven articles were classified as awaiting assessment because eligibility could not be adequately evaluated given available information (Aracena *et al*., 2011; Aracena *et al*., 2012; Akbarzadeh *et al*., 2016; Shirazi *et al*., 2016; Frith *et al*., 2017; Kahalili *et al*., 2019; Tran *et al*., 2019). The most common reasons for exclusion were studies that described an intervention that did not aim to improve MMH and studies that did not include a child outcome (Fig. 1). The 36 included articles represent data from 21 RCTs and 28 284 mother–child dyads.

### Overview of study characteristics and quality

Population: most trials were conducted in upper-middle-income countries (Brazil, Chile, China, Iran, Lebanon, Malaysia, Mexico and South Africa) (Langer *et al*., 1998; Bastani *et al*., 2006; Aracena *et al*., 2009; Carvalho *et al*., 2009; Cooper *et al*., 2009; Le Roux *et al*., 2013; Le Roux *et al*., 2014; Rotheram-Borus *et al*., 2014a; Rotheram-Borus *et al*., 2014b; Tomlinson, 2014; Karamoozian and Askarizadeh, 2015; Murray *et al*., 2015; Tomlinson *et al*., 2015; Tomlinson *et al*., 2016b; Tomlinson *et al*., 2016a; Zhao *et al*., 2017, Rotheram-Fuller *et al*., 2018, Tomlinson *et al*., 2018; Mohd Shukri *et al*., 2019; Nabulsi *et al*., 2019; Rotheram-Borus *et al*., 2019; Guo *et al*., 2020; Zhao *et al*., 2020) followed by lower-middle-income (India, Nigeria and Pakistan) (Rahman *et al*., 2008; Tripathy *et al*., 2010; Maselko *et al*., 2015; Dabas *et al*., 2019; Fuhr *et al*., 2019; Gureje *et al*., 2019; Sikander *et al*., 2019; Rajeswari and SanjeevaReddy, 2020), low-income (Pakistan) (Rahman *et al*., 2008; Maselko *et al*., 2015) and a multi-site trial of lower-middle (Cuba) and upper-middle-income countries (Argentina, Brazil and Mexico) (Villar *et al*., 1992) (Table 1). Most trials enrolled pregnant women in their second and/or third trimester (Villar *et al*., 1992; Langer *et al*., 1998; Bastani *et al*., 2006; Rahman *et al*., 2008; Aracena *et al*., 2009; Cooper *et al*., 2009; Le Roux *et al*., 2013; Le Roux *et al*., 2014; Tomlinson, 2014; Rotheram-Borus *et al*., 2014a; Rotheram-Borus *et al*., 2014b; Maselko *et al*., 2015; Murray *et al*., 2015; Tomlinson *et al*., 2015; Tomlinson *et al*., 2016a; Tomlinson *et al*., 2016b; Fuhr *et al*., 2019; Gureje *et al*., 2019; Kola *et al*., 2019; Mohd Shukri *et al*., 2019; Oladeji *et al*., 2019; Sikander *et al*., 2019; Guo *et al*., 2020; Rajeswari and SanjeevaReddy, 2020; Zhao *et al*., 2020). Four trials enrolled women that had recently given birth (Carvalho *et al*., 2009; Tripathy *et al*., 2010; Le Roux *et al*., 2013; Le Roux *et al*., 2014; Rotheram-Borus *et al*., 2014b; Tomlinson, 2014; Tomlinson *et al*., 2015; Tomlinson *et al*., 2016a; Tomlinson *et al*., 2016b, Rotheram-Fuller *et al*., 2018, Tomlinson *et al*., 2018; Dabas *et al*., 2019; Rotheram-Borus *et al*., 2019). Some trials enrolled specific subgroups of pregnant women including adolescents or young adults (Aracena *et al*., 2009), low-income (Cooper *et al*., 2009; Le Roux *et al*., 2013; Le Roux *et al*., 2014; Rotheram-Borus *et al*., 2014b; Tomlinson, 2014; Murray *et al*., 2015; Tomlinson *et al*., 2015; Tomlinson *et al*., 2016a; Tomlinson *et al*., 2016b, Rotheram-Fuller *et al*., 2018, Tomlinson *et al*., 2018; Rotheram-Borus *et al*., 2019), pregnant with a single foetus, no previous vaginal delivery and no evidence of severe obstetric disease (Langer *et al*., 1998; Mohd Shukri *et al*., 2019), high-risk pregnancies (Zhao *et al*., 2017) or HIV-positive (Rotheram-Borus *et al*., 2014a). Several trials enrolled subgroups of pregnant women meeting specific mental health criterion including having mild to moderate stress (Rajeswari and SanjeevaReddy, 2020), elevated anxiety or depressive symptoms (Bastani *et al*., 2006; Cooper *et al*., 2009; Murray *et al*., 2015; Guo *et al*., 2020) screening positive for depression based on PHQ9 ≥ 10 (Fuhr *et al*., 2019), EPDS ≥ 9 (Zhao *et al*., 2017; Zhao *et al*., 2020), EPDS = 12 (Karamoozian and Askarizadeh, 2015), anxiety based on the Pregnancy-Related Anxiety Questionnaire (PRAQ) (Karamoozian and Askarizadeh, 2015) or DSM-IV-TR criteria for major depressive episode (Rahman *et al*., 2008; Maselko *et al*., 2015).

**Table 1.** Summary of included studies

Study	Setting	Intervention	Control group	Target population	Child development outcome(s)	Timing of assessment(s)	Number of participants		
							*N*	Exp.	Con
Aracena et al. (2009)	Santiago, Chile	Home visits	Standard care (on average 10 prenatal visits and well-baby visits)	Adolescent primiparous women treated in 1 of 2 health centres in a low-income neighbourhood	Malnutrition (underweight, overweight), psychomotor development, Incidence of illness	Pre- and post-intervention (12–15 months)	90	45	45
Bastani et al. (2006)	Tehran, Iran	Applied relaxation training	Standard care (routine, hospital-based prenatal care)	Primiparous women in second trimester with high levels of anxiety	Low birth weight, average weight, preterm birth, instrumental delivery	Post-intervention (7 weeks)	110	55	55
Carvalho et al. (2009)	Sao Paulo, Brazil	Clinic-based psychological support and video and manual support materials	Standard care (clinic-based psychological support)	Mothers of preterm newborns and very low birth weights, hospitalised in a Neonatal Intensive Care Unit	Neonatal period (duration of hospitalisation in NICU (days), total duration of hospitalisation (days))	Post-intervention (end of hospitalisation)	59	36	23
Cooper et al. (2009); Murray et al. (2015)	Cape Town, South Africa	Home visits	Standard care (home visits every 2 weeks)	Pregnant women in 3rd trimester with high levels of depression in two areas of a peri-urban settlement (Khayelitsha) in Cape Town	Attachment style; cognitive development	18 months after birth	449	220	229
Dabas et al. (2019)	New Delhi, India	Audio-assisted relaxation technique	Standard care (hospital-based care)	Postpartum mothers whose children were born preterm	Milk output	10 days after birth	57	29	28
Fuhr et al. (2019)	Goa, India	Thinking Healthy Program, peer-delivered	Enhanced usual care from gynaecologist who received depression screening results and mhGAP	Pregnant women in their 2nd or 3rd trimester receiving antenatal care	Exclusive breastfeeding; weight-for-age; height-for-age	3 and 6 months	280	140	140
Guo et al. (2020)	Tianjin, China	Mindful self-compassion programme	Wait-list control group	Pregnant women in their 2nd or 3rd trimester at high risk of post-partum depression	Infant temperament	3 and 12 months	314	157	157
Gureje et al. (2019); Kola et al. (2019); Oladeji et al. (2019)	Oyo State, Nigeria	Problem solving treatment	Enhanced usual care (low-intensity treatment)	Pregnant women in their 2nd or 3rd trimester with major depressive disorder and seeking care in an enrolled primary maternal care clinic	Infant growth; infant health; exclusive breastfeeding; motor and cognitive development	6 months	686	452	234
Karamoozian et al. (2015)	Kerman, Iran	Cognitive behavioural stress management	Standard care (clinic-based prenatal care)	Pregnant women with anxiety and depression, referring to three health clinics in Kerman	Apgar score (infant health status)	1 min and 5 min after birth	30	15	15
Langer et al. (1998)	Mexico City, Mexico	Psychosocial support from doula during labour	Standard care (not described)	Pregnant women with a single foetus, no previous vaginal delivery and no evidence of severe obstetric disease who arrived at the labour and delivery unit	Excusive breastfeeding; full breastfeeding	1 month post-partum	724	361	363
Le Roux et al. (2013, 2014); Rotheram-Borus et al. (2014b, 2019a, 2019b); Rotheram-Fuller et al. (2018); Tomlinson et al. (2014, 2015, 2016a, 2016b, 2018); Christodoulou et al. (2019)	Cape Town, South Africa	Home visits	Standard care (access to healthcare at government clinics and hospitals)	Pregnant women living in townships surrounding Cape Town and obtaining care at government clinics	Birth weight, weight-for-age, height-for-age, weight-for-height, head circumference, exclusive breastfeeding, child health status, growth/development, diarrhoea, language, clinic visits	Post-birth, 6-months, 18-months and 36-months	1238	644	594
Maselko et al. (2015); Rahman et al. (2008)	Punjab Province, Pakistan	Home visits; Thinking Healthy Program	Enhanced usual care – antenatal and postnatal services by community health workers	Married pregnant women with prenatal depression in rural Pakistan	Cognitive development; socioemotional development; physical development (height-for-age, weight-for-age, BMI-for-age) stunting (height-for-age); underweight (weight-for-age); exclusive breastfeeding	7 years (Maselko) 6 months; 12 months (Rahman)	705 903	360 463	345 440
Mohd Shukri et al. (2019)	Klang-Valley, Malaysia	Relaxation audio therapy and home visits	No intervention	First time pregnant women in their 2nd or 3rd trimester attending antenatal clinics who deliver a healthy, full-term infant and were exclusively breastfeeding	Infant behaviour; anthropometry; milk intake	2–6 and 12 weeks	64	33	31
Nabulsi et al. (2019)	Beirut, Lebanon	Multicomponent breastfeeding support intervention	Standard obstetric and paediatric care	Healthy pregnant women in their 1st or 2nd trimester seeking antenatal care	Exclusive breastfeeding	1, 3 and 6 months	446	222	224
Rajeswari & Sanjeevareddy (2020)	Chennai, India	Progressive muscle relaxation	Standard antenatal care	Pregnant women in their 2nd trimester with minimal to moderate stress levels	Foetal/newborn complications; birthweight	Birth; 10 weeks	250	125	125
Rotheram-Borus et al. (2014a)	KwaZulu-Natal, South Africa	Clinic-based psychological support	Standard care (prevention of maternal-to-child HIV transmission services)	Pregnant women testing positive for HIV in 1 of 8 clinics in KwaZulu Natal	Exclusive breastfeeding; weight-for-age; height-for-age; weight-for-height; postpartum bonding; normal development (50th percentile)	1.5 months, 6 months and 12 months post-partum	1200	544	656
Sikander et al. (2019)	Rawalpindi, Pakistan	Thinking Health Program, peer-delivered	Enhanced usual care by doctors/midwives who were given screening results and mhGAP perinatal depression treatment guidelines	Pregnant women in their 3rd trimester with depressive symptoms	Exclusive breastfeeding; infant growth	3 and 6 months	494	283	211
Tripathy et al. (2010)	Jharkhand and Orissa Districts, India	Participatory Women's Groups	Health committee focused on health services	Women who had just given birth	Neonatal deaths and mortality rate; stillbirths	1, 2 and 3 years post-partum	19,140	9770	9260
Villar et al. (1992)	Latin America (Rosario, Argentina; Pelotas, Brazil; Havana, Cuba; Mexico City, Mexico)	Home visits	Standard care (clinic-based routine prenatal care)	Women at high risk for delivery a low birth weight infant and are less than 20 weeks pregnant	Low birth weight, incidence of infant morbidity (respiratory infection, gastrointestinal disease, nutritional complications, diarrhoea, dehydration), pregnancy outcomes	Birth, 40 days postpartum	2235	1115	1120
Zhao et al. (2017)	Shanghai, China	Psychoeducational programme for first-time parents	Standard care (routine obstetrical care)	Women with high-risk pregnancies and obstetric complications and at high-risk for postpartum depression	Breastfeeding; infant growth; infant sleep	42 days postpartum	352	176	176
Zhao et al. (2020)	Shanghai, China	Individualised mixed management intervention	Standard obstetric care	Primiparous pregnant women in their 3rd trimester with depressive symptoms	Exclusive breastfeeding	3 days postpartum	182	91	91

Exp, experimental group; Con, control group.

Interventions (Table 2): nine trials delivered the intervention through home visits provided by health educators (Aracena *et al*., 2009), peers (Fuhr *et al*., 2019; Sikander *et al*., 2019), nurses (Villar *et al*., 1992), certified lactation consultants (Nabulsi *et al*., 2019), community health workers (Rahman *et al*., 2008; Cooper *et al*., 2009; Le Roux *et al*., 2013; Rotheram-Borus *et al*., 2014b; Tomlinson, 2014; Maselko *et al*., 2015; Murray *et al*., 2015; Tomlinson *et al*., 2015; Le Roux *et al*., 2014; Tomlinson *et al*., 2016a; Tomlinson *et al*., 2016b; Rotheram-Borus *et al*., 2019, Rotheram-Fuller *et al*., 2018, Tomlinson *et al*., 2018), social workers (Villar *et al*., 1992) or a researcher (Mohd Shukri *et al*., 2019). Twelve trials delivered the intervention in hospital- or clinic-based settings by nurse researchers (Bastani *et al*., 2006), primary maternal care providers (Gureje *et al*., 2019), psychologists (Carvalho *et al*., 2009), doulas/midwives/lactation consultants (Langer *et al*., 1998; Nabulsi *et al*., 2019; Zhao *et al*., 2020), peers (Rotheram-Borus *et al*., 2014a; Fuhr *et al*., 2019; Sikander *et al*., 2019) or research staff (Zhao *et al*., 2017; Dabas *et al*., 2019; Mohd Shukri *et al*., 2019; Rajeswari and SanjeevaReddy, 2020). One of these interventions was delivered online (Guo *et al*., 2020), and several supplemented in-person activities with audio/video materials (Carvalho *et al*., 2009; Dabas *et al*., 2019; Mohd Shukri *et al*., 2019). One of these clinic-based trials did not specify the provider (Karamoozian and Askarizadeh, 2015). The final trial delivered the intervention in community-based settings via a local female facilitator (Tripathy *et al*., 2010). The majority of interventions were child-focused, but all contained an MMH component.

**Table 2.** Intervention details

Study	Total number of sessions (timing, session duration)	Number of sessions focused on mental health	Content of mental health sessions	Content of other sessions	Types of facilitators	Delivery mode	Intervention timing
Aracena et al. (2009)	On average 12 (1-h sessions over a year)	NR	Education and activities surrounding self-esteem and problem solving	Educational home visits covering topics on identity, goals, child care	Trained health educators supervised by a nurse-midwife	Individual; home-based	Pregnancy and early postpartum period
Bastani et al. (2006)	7 (weekly, 90 min)	7	Entire intervention was relaxation training to reduce anxiety	N/A	Nurse researcher trained by a clinical psychologist	Group education sessions; clinic-based	2nd trimester
Carvalho et al. (2009)	2 (not reported)	2	Psychological guidance for mothers of preterm babies	N/A	Psychologist	Clinic-based psychological support groups; video and manual support materials	Infant's hospitalisation
Cooper et al. (2009); Murray et al. (2015)	16 (visited, ideally, twice antenatally, weekly for the first 8 weeks postpartum, fortnightly for a further 2 months, and then monthly for 2 months, 1-h visits)	16	Counselling and psychological support; promoting secure attachment	N/A	Lay community workers trained in intervention manual	Individual; home-based	3rd trimester to 6-months post-partum
Dabas et al. (2019)	10 (daily sessions, 30 minutes)	10	Relaxation exercises including deep breathing, Suksham Vyayam, Anulom-Vilom), Brahmari, Progressive muscle relaxation, and deep breathing	N/A	Audio recording, supervised by researcher	Audio recording on a laptop; hospital-based	Post-partum
Fuhr et al. (2019)	6–14 (30–45 min)	6–14	Cognitive behavioural therapy with a focus on strategies that incorporate behavioural activation	N/A	Peers selected based on their interest in helping/supporting other women in their community, and had good communication skills	Individual, home- based	Prenatal to 6 months postpartum
Guo et al. (2020)	36 (6 episodes per week, 15 min)	36	Mindfulness and self-compassion	N/A	Online-based	Online-based	Pregnancy
Gureje et al. (2019); Kola et al. (2019); Oladeji et al. (2019)	12–16 (8 weeks prenatal, weekly or fortnightly postnatal, 30–45 min)	12–16	Problem–solving treatment including breaking down current stressors and exploring options for resolving these problems	N/A	Primary maternal care provider	Individual, primary maternal care clinic	Prenatal to 8 weeks postnatal
Karamoozian et al. (2015)	12 (12 weekly, 90 min)	12	Stress-coping techniques including an introduction to stressors and stress responses, relationship between emotions and thoughts, negative thinking, relaxation training, correction of cognitive distortions, training of effective coping responses and anger management	N/A	Not stated	Group training sessions; clinic-based	Pregnancy
Langer et al. (1998)	1 (NR)	1	Psychosocial support	N/A	Doula	Individual; hospital-based	Labour through immediate post-partum period
Le Roux et al. (2013, 2014); Rotheram-Borus et al. (2014b, 2019a, 2019b); Rotheram-Fuller et al. (2018); Tomlinson et al. (2014, 2015, 2016a, 2016b, 2018); Christodoulou et al. (2019)	On average, 6 antenatal visits (range 1–27), 5 postnatal visits (range 1–12) (about 1.4 sessions a month lasting 31 min on average)	NR	Cognitive behavioural change strategies; teaching problem solving, infant bonding and alcohol use prevention/reduction	Perinatal home visits to teach nutrition, HIV testing/prevention and growth monitoring	Trained community health workers	Individual; home-based	Pregnancy through 6-months post-partum
Maselko et al. (2015); Rahman et al. (2008)	16 (a session in the last month of pregnancy, three sessions in the first postnatal month, nine 1-monthly sessions thereafter; session duration NR)	16	Cognitive behavioural intervention aimed at improving ‘positive and healthy thinking’	N/A	Community health workers	Individual; home-based	3rd trimester through 10-months post-partum
Mohd Shukri et al. (2019)	4 (every 2-4 weeks, 2–3 h)	4	Relaxation therapy tape to be used during breastfeeding or milk expression that involved guided imagery designed for breastfeeding mothers	Mothers also received standard breastfeeding support, a guidance booklet, and a list of resources	Researcher	Individual; home- based and audio tape	Post-partum
Nabulsi et al. (2019)	Minimum of 24 (10 scheduled calls or hospital/home visits starting with the antenatal class, then at 6 and 9 months gestation, expected week of delivery, first day postpartum, 48 h post hospital discharge, 1, 2, 4 weeks postpartum, and monthly thereafter until 6 months postpartum)	Minimum of 24	Emotional support and strengthening social capital	Prenatal breastfeeding education, postpartum professional lactation support to improve maternal self-efficacy	Certified lactation consultant and support mothers who had successfully breastfed at least one child for a minimum of 2 months and had positive breastfeeding attitudes	Individual; combination of hospital visits, home visits and scheduled calls	Pregnancy to 6-months post-partum
Rajeswari & Sanjeevareddy (2020)	2 (daily, 20–25 min) followed by 10 weeks of practice	2	Progressive muscle relaxation and deep breathing	N/A	Researcher	Hospital- based, outpatient	Pregnancy
Rotheram-Borus et al. (2014a)	8 (4 antenatal, 4 postnatal)	NR	Education about establishing healthy routines without alcohol and smoking, building and maintaining a social network, bonding with the infant and normalising being a women with HIV	Counselling on adherence to recommended HIV care, obtaining a child support grant, condom use and preventing HIV transmission and HIV testing and disclosure	HIV-positive peer mentors trained in cognitive-behavioural skills	Clinic-based	Pregnancy – 6-months post-partum
Sikander et al. (2019)	14 (10 sessions delivered during pregnancy and in first 3 months after childbirth 30–45 min)	14	Cognitive behavioural therapy with a focus on strategies that incorporate behavioural activation	N/A	Peers who were local volunteers, married, 30–35 years, and had displayed communication skills	10 individual sessions delivered at participants’ homes; 4 group sessions delivered at community health facilities or convenient location	Pregnancy – 6-months post-partum
Tripathy et al. (2010)	20 (monthly sessions, duration NR)	NR	Social support, problem-solving skills, discussion of MMH challenges	Identifying and prioritising maternal and newborn health problems; identifying strategies to address these problems and discussion their effects	Local female facilitator identified by community members	Community-based	Monthly meetings
Villar et al. (1992)	4 (approximately every 4 weeks, 1–2 h)	4	Psychosocial/emotional support and decision-making	Promoting healthy behaviour; encouraging prenatal care visit attendance; health education	Social workers, nurses	Individual; home-based	22–34 weeks gestation
Zhao et al. (2017)	6 (Timing NR, 1.5 hours)	5	Antenatal anxiety, postpartum depression, coping skills, encouraging psychological adjustment	High-risk pregnancy knowledge, supportive husbands	Research staff	Group-based; husband joined for 6th session; hospital-based	Pregnancy (enrolled before 28 weeks gestation)
Zhao et al. (2020)	4 (following antenatal care appointments, 60 min)	4	Psychoeducation	Breastfeeding methods and coping skills for breastfeeding problems	First 3 sessions led by midwives and the last session provided by a lactation consultant	Clinic-based	Pregnancy

MMH components included education surrounding self-esteem and/or problem-solving (Aracena *et al*., 2009; Tripathy *et al*., 2010; Le Roux *et al*., 2013; Le Roux *et al*., 2014; Tomlinson, 2014; Rotheram-Borus *et al*., 2014b; Tomlinson *et al*., 2015; Tomlinson *et al*., 2016a; Tomlinson *et al*., 2016b; Gureje *et al*., 2019; Oladeji *et al*., 2019), strengthening social networks (Rotheram-Borus *et al*., 2014a, Rotheram-Fuller *et al*., 2018, Tomlinson *et al*., 2018; Nabulsi *et al*., 2019; Rotheram-Borus *et al*., 2019), provision of social, psychological and emotional support (Villar *et al*., 1992; Cooper *et al*., 2009; Tripathy *et al*., 2010; Murray *et al*., 2015; Nabulsi *et al*., 2019), cognitive-behavioural strategies (Le Roux *et al*., 2013; Le Roux *et al*., 2014; Rotheram-Borus *et al*., 2014a; Rotheram-Borus *et al*., 2014b; Tomlinson, 2014; Tomlinson *et al*., 2015; Karamoozian and Askarizadeh, 2015; Tomlinson *et al*., 2016a; Tomlinson *et al*., 2016b, Rotheram-Fuller *et al*., 2018, Tomlinson *et al*., 2018; Fuhr *et al*., 2019; Rotheram-Borus *et al*., 2019; Sikander *et al*., 2019), alcohol use prevention (Le Roux *et al*., 2013; Rotheram-Borus *et al*., 2014b; Tomlinson, 2014; Rotheram-Borus *et al*., 2014a; Tomlinson *et al*., 2015, Rotheram-Fuller *et al*., 2018, Tomlinson *et al*., 2018; Rotheram-Borus *et al*., 2019), relaxation techniques (Karamoozian and Askarizadeh, 2015; Dabas *et al*., 2019; Mohd Shukri *et al*., 2019; Rajeswari and SanjeevaReddy, 2020), mindfulness (Guo *et al*., 2020) and psychoeducation (Zhao *et al*., 2017; Zhao *et al*., 2020). The primary aim of 11 trials was focused specifically on improving MMH via interventions that included relaxation and mindfulness training to reduce anxiety/stress (Bastani *et al*., 2006; Rajeswari and SanjeevaReddy, 2020) or depression (Guo *et al*., 2020), cognitive-behavioural or problem solving therapy to reduce depressive symptoms (Rahman *et al*., 2008; Maselko *et al*., 2015; Fuhr *et al*., 2019; Gureje *et al*., 2019; Sikander *et al*., 2019), psychoeducation to reduce depression and anxiety (Zhao *et al*., 2017) and provision of psychological or social support to reduce depression, anxiety or stress (Villar *et al*., 1992; Langer *et al*., 1998; Carvalho *et al*., 2009).

Child outcomes: nutrition and growth outcomes included exclusive breastfeeding (Langer *et al*., 1998; Rahman *et al*., 2008; Le Roux *et al*., 2013; Le Roux *et al*., 2014; Rotheram-Borus *et al*., 2014a; Rotheram-Borus *et al*., 2014b; Maselko *et al*., 2015; Tomlinson *et al*., 2015; Tomlinson *et al*., 2016a; Tomlinson *et al*., 2016b; Zhao *et al*., 2017; Fuhr *et al*., 2019; Gureje *et al*., 2019; Nabulsi *et al*., 2019; Sikander *et al*., 2019; Rajeswari and SanjeevaReddy, 2020; Zhao *et al*., 2020), low birth weight (Villar *et al*., 1992; Bastani *et al*., 2006; Le Roux *et al*., 2013; Rajeswari and SanjeevaReddy, 2020) and nutritional status and child growth (e.g. weight-for-age, height-for-age and weight-for-height) (Rahman *et al*., 2008; Aracena *et al*., 2009; Le Roux *et al*., 2013; Le Roux *et al*., 2014; Rotheram-Borus *et al*., 2014a; Rotheram-Borus *et al*., 2014b; Maselko *et al*., 2015; Tomlinson *et al*., 2015; Tomlinson *et al*., 2016a; Tomlinson *et al*., 2016b; Tomlinson *et al*., 2018; Fuhr *et al*., 2019; Gureje *et al*., 2019; Mohd Shukri *et al*., 2019; Rotheram-Borus *et al*., 2019; Sikander *et al*., 2019). Child development outcomes were assessed between birth and 84-months post-partum. Psychomotor or cognitive development (Aracena *et al*., 2009; Rotheram-Borus *et al*., 2014a; Maselko *et al*., 2015; Murray *et al*., 2015; Tomlinson *et al*., 2018; Rotheram-Borus *et al*., 2019) were measured using the Psychomotor Development Scale (Rodriguez *et al*., 1974), the Wechsler Preschool and Primary Scale of Intelligence (WPPSI-IV) (Wechsler, 1989), the Strengths and Difficulties Questionnaire (SDQ) (Syed *et al*., 2007), the Spence Children's Anxiety Scale (SCAS) (Spence, 1998), the Bayley Scales (version II) (Bayley, 1993) and the World Health Organization (WHO) gross motor milestones (Wijnhoven *et al*., 2004). Two trials focused on the mother–child relationship: one trial assessed attachment style (Cooper *et al*., 2009) and one trial measured postpartum bonding (Rotheram-Borus *et al*., 2014a). Several trials also assessed the incidence of infant morbidities and mortality (Villar *et al*., 1992; Aracena *et al*., 2009; Tripathy *et al*., 2010; Gureje *et al*., 2019; Rajeswari and SanjeevaReddy, 2020); however, outcome definitions varied substantially between trials. Other outcomes, which were measured in a single trial, include head circumference-for-age (Le Roux *et al*., 2013) and number of days in the neonatal intensive care unit (Carvalho *et al*., 2009).

### Risk of bias and GRADE certainty of evidence

**Fig. 2:**
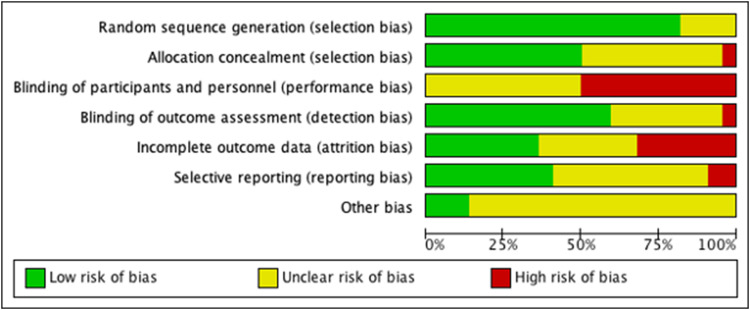
**.** Risk of bias in included studies.

Few studies showed high risk of bias on two or more of the seven domains assessed in this review. While all included trials were RCTs, three trials did not describe how the randomisation sequence was generated leading to unclear risk of bias. Similarly, the method of allocation concealment was not well described in eight trials. Only ten trials described acceptable methods for blinding outcome assessors. Attrition and selective outcome reporting were common sources of bias that could compromise the validity of trials (Fig. 2). Certainty of evidence ranged from very low to high using the GRADE methodology. Downgrading was due to the high level of heterogeneity across studies (i.e. *I*^2^ above 55%), lack of information on masking of outcome assessors and attrition (online Supplementary File 1).

### Narrative synthesis and meta-analyses

A summary of the results from meta-analyses is provided in Table 3. Growth indicators: the earliest growth indicator, low birth weight, was reported in four publications representing three trials. Findings were inconclusive as one trial reported a lower prevalence of low birth weight in infants of mothers in the intervention *v*. control (Bastani *et al*., 2006), while others found marginal (Le Roux *et al*., 2013; Rotheram-Borus *et al*., 2014b) or no difference in the prevalence of low birth weight between groups (Villar *et al*., 1992) (online Supplementary File 2).

**Table 3.** Summary of quantitative synthesis

Outcome	Outcome type	Number of trials	Participants	Pooled effect estimate
Exclusive breastfeeding	Dichotomous	10	4749	RR = 1.39 (95% CI: 1.13–1.71)
Low birthweight	Dichotomous	3	3243	RR = 0.73 (95% CI: 0.47–1.12)
Not stunted (HAZ ≥ −2)	Dichotomous	3	1880	RR = 1.02 (95% CI: 0.97–1.08)
Not underweight (WAZ ≥ −2)	Dichotomous	4	2505	RR = 1.00 (95% CI: 0.99–1.02)
Weight for height ≥−2	Dichotomous	2	1151	RR = 0.92 (95% CI: 0.77–1.10)
Weight	Continuous	5	1707	SMD = 0.16 (95% CI: −0.05 to 0.36)
Height	Continuous	3	1388	SMD = 0.13 (95% CI: 0.02–0.24)
Psychomotor development	Continuous	2	496	SMD = 0.05 (95% CI: −0.13 to 0.23)
Cognitive development	Continuous	3	1256	SMD = 0.07 (95% CI: −0.04 to 0.18)

Standardised measures of weight-for-age and height-for-age were evaluated in five trials. Three trials reported weight- or height-for-age on a continuous scale (Rahman *et al*., 2008; Fuhr *et al*., 2019; Sikander *et al*., 2019). The observed effect of the intervention on greater height-for-age in the trial by Rahman and colleagues (2008) was nullified after adjusting for baseline covariates at 6- and 12-months. However, the pooled effect of three trials of the Thinking Healthy Program found a small effect of the intervention on greater height-for-age at 6 months (SMD = 0.13, 95% confidence interval (CI): 0.02–0.24; online Supplementary File 3). Two additional trials measured weight on a continuous scale (Mohd Shukri *et al*., 2019; Rajeswari and SanjeevaReddy, 2020), and when combined with the three Thinking Health Program trials, we did not find an effect of these interventions on child weight (online Supplementary File 4).

Several publications transformed height-for-age and weight-for-age into a binary variable indicating whether a child was stunted or underweight (Rahman *et al*., 2008; Aracena *et al*., 2009; Le Roux *et al*., 2013; Rotheram-Borus *et al*., 2014a; Tomlinson *et al*., 2015). Le Roux *et al*. (2013) found that infants in the home visit intervention group were less likely to be stunted at 6-months, but found no between-group differences for underweight. Tomlinson and colleagues found that infants of depressed mothers in the intervention group were comparable to infants of non-depressed mothers under intervention and control conditions in terms of height-for-age; whereas, infants of depressed mothers under control conditions had lower height-for-age at 6-months. Weight-for-age did not differ by condition or maternal depression (Tomlinson *et al*., 2015). At 18-months, there was no difference in the odds of stunting between intervention conditions among children of mothers with elevated symptoms of antenatal depression, yet the odds of being underweight were greater under control conditions (Tomlinson *et al*., 2018). In the same trial, weight-for-height findings were complex: children of depressed mothers under intervention conditions were at WHO recommended weight-for-height scores (i.e. weight-for-height *z*-score = 0), but children of non-depressed mothers (intervention and control conditions) and children of depressed mothers under control conditions, were above WHO recommended weight-for-height scores (i.e. weight-for-height *z*-score > 0). The authors suggest that these findings can be explained by the intervention children being taller and less likely to be stunted, whereas children of depressed mothers under control conditions were shorter and similar in weight to children of depressed mothers under intervention conditions and children of non-depressed mothers under intervention and control conditions (Tomlinson *et al*., 2015). A separate trial that identified a main effect of the intervention on the odds of not being underweight (odds ratio (OR) = 1.08, 95% CI: 1.01–1.16), but no intervention effects on stunting from birth to 12-months (OR = 0.99, 95% CI: 0.90–1.08) (Rotheram-Borus *et al*., 2014a). Meta-analyses of categorical growth indicators did not find evidence of pooled intervention effects for being underweight, being stunted, and severe acute malnutrition – weight-for-height (online Supplementary Files 5–7).

Child health status: newborn health status was reported in seven trials and was operationalised as a function of growth and development indicators (Rotheram-Borus *et al*., 2014b), infant/foetal complications (Rajeswari and SanjeevaReddy, 2020), incidence of illness (Aracena *et al*., 2009; Gureje *et al*., 2019), Apgar score (Karamoozian and Askarizadeh, 2015; Rajeswari and SanjeevaReddy, 2020), duration of hospitalisation (Carvalho *et al*., 2009) or neonatal mortality (Tripathy *et al*., 2010). Heterogeneity in outcome definitions precluded meta-analysis of child health status, but independent studies reported positive intervention effects on Apgar scores and neonatal mortality (Tripathy *et al*., 2010; Karamoozian and Askarizadeh, 2015). In contrast, one study found that psychological intervention was associated with more hospital and NICU days, mixed findings related to postpartum complications (Rajeswari and SanjeevaReddy, 2020), and no effect of interventions on the incidence of child illness (Aracena *et al*., 2009; Carvalho *et al*., 2009; Gureje *et al*., 2019).

**Fig. 3:**
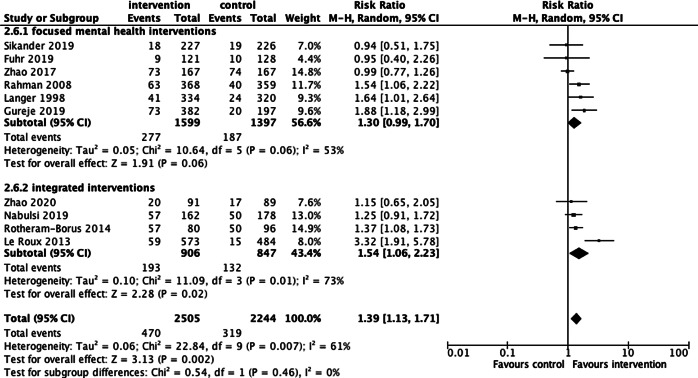
**.** Exclusive breastfeeding by type of mental health intervention (focused *v*. integrated).

Breastfeeding: ten trials included breastfeeding as an outcome. Results of the meta-analysis (*n* = 4749) including data across ten comparisons indicated a sizeable overall impact in favour of intervention with moderate certainty according to the GRADE assessment: RR of 1.39, 95% CI: 1.13–1.71, NNT = 22.00, 95% CI: 15.00–40.90) (Langer *et al*., 1998; Rahman *et al*., 2008; Le Roux *et al*., 2013; Rotheram-Borus *et al*., 2014a; Zhao *et al*., 2017; Fuhr *et al*., 2019; Gureje *et al*., 2019; Nabulsi *et al*., 2019; Sikander *et al*., 2019; Zhao *et al*., 2020) (Fig. 3). Heterogeneity was significant (*I*^2^ = 61%) indicating substantial variation between interventions in their impacts on the outcome. Sub-group analyses revealed slightly larger effect sizes for integrated MMH interventions compared to focused MMH interventions, however uncertainty was high in these subgroups.

Maternal–child relationship outcomes: one trial focusing on the mother–child relationship found more secure attachment of infants of mothers under the intervention relative to the control conditions (74 *v*. 63%), which was driven by a higher probability of avoidant attachment in control infants (19 *v*. 11%) (Cooper *et al*., 2009). In contrast, results from another trial found the proportion of infants with ‘normal bonding’ similar under intervention (98%) and control (98.9%) conditions (Rotheram-Borus *et al*., 2014a).

Developmental outcomes: seven publications representing four trials evaluated one or more of the following domains of child development: cognitive development, language development, socio-emotional development, motor development, physical development, aggressive and prosocial behaviour and executive functioning. When evaluating development as one broad outcome, there were no differences between infants of mothers under the intervention relative to the control conditions in the short- and long-term (Aracena *et al*., 2009; Rotheram-Borus *et al*., 2014a; Rotheram-Borus *et al*., 2014b; Maselko *et al*., 2015). Results focusing on specific domains of child development were mixed (Aracena *et al*., 2009; Maselko *et al*., 2015; Murray *et al*., 2015). Cognitive and psychomotor developments were the only indicators measured in more than one study. We did not observe an impact of MMH interventions on cognitive development (3 trials, 1256 participants, SMD = 0.07, 95% CI: −0.04 to 0.18, *I*^2^ = 0%; online Supplementary File 8) (Maselko *et al*., 2015; Murray *et al*., 2015; Tomlinson *et al*., 2018). Similarly, there was no effect of MMH interventions on psychomotor development (2 trials, 496 participants, SMD = 0.05, 95% CI: −0.13 to 0.23, *I*^2^ = 0%; online Supplementary File 9) (Aracena *et al*., 2009; Le Roux *et al*., 2013).

## Discussion

The aim of this systematic review and meta-analysis was to summarise existing experimental knowledge regarding the impact of MMH interventions on child-related outcomes. We identified 21 RCTs reporting on more than 28 000 participants. All trials focused on common mental disorders and most were conducted in middle-income countries.

The most commonly included outcome across these trials was exclusive breastfeeding. A recent meta-analysis found breastfeeding to be protective against child infections and malocclusion, associated with higher intelligence, and probable reductions in overweight and diabetes (Victora *et al*., 2016). Nevertheless, only 37% of children under 6-months are exclusively breastfed in LMICs (Victora *et al*., 2016). In our study, meta-analysis of ten comparisons with a combined number of 4749 women showed that with intervention 39% more children are exclusively breastfed than under control conditions.

Given the varied nature of the interventions, it is challenging to single out the unique influence of the MMH components on improved rates of exclusive breastfeeding. However, one broad observation supports the contribution that MMH components can make in improving rates of exclusive breastfeeding. Future studies can be improved in two ways to clarify the impact of mental health components on exclusive breastfeeding. First, trials could be designed specifically so that mediation analyses can be conducted to assess whether improvements in MMH are in turn associated with exclusive breastfeeding. Second, head-to-head comparisons of interventions with and without a mental health component would be helpful to estimate the additional contribution of MMH components in integrated interventions.

Meta-analyses on other outcomes did not identify sizeable benefits of intervention and there was high heterogeneity between studies. These meta-analyses were limited by fewer available publications relative to the exclusive breastfeeding meta-analysis and should be interpreted with caution. There was a significant pooled effect of intervention on child height, but the effect size was small and only incorporated findings from three trials. There were trends favouring intervention for cognitive and psychomotor development, low birth weight, weight-for-age and height-for-age, but these did not reach statistical significance. It is possible that MMH interventions may have impacts on particular development domains, but not on broad indicators of child development. Similarly, MMH interventions may have impacts on particular growth indicators at specific developmental stages.

Before discussing implications of this systematic review and meta-analysis, we note the strengths and limitations of the existing literature. Overall, few trials included in this systematic review showed high risk of bias. Attrition and lack of masking were the greatest sources of potential bias. We presented conservative intention-to-treat analyses, but attrition introduced significant uncertainty in estimates. A substantive limitation to the generalisability of this review is that all interventions were focused on common mental disorder. It would be helpful for future studies to also evaluate whether interventions for other mental health outcomes (e.g. psychosis) are associated with improvements in child-related outcomes. Additionally, only one trial was conducted in a low-income country. Scaling of interventions may be particularly challenging in such settings, so further studies assessing impacts in low-income countries would be useful. Finally, there was substantial variation in how outcomes were defined and assessed, which limited the possibility to conduct meta-analyses for some outcomes.

Results from this review should be considered in light of several limitations in the review process. First, we included trials with diverse populations, who may respond differently to MMH interventions. Second, we included child outcomes that were reported across different studies, but did not prespecify primary *v*. secondary outcome measures, increasing the risk for selective reporting. However, we attempted to report on all available outcomes, without focusing only on those that were included in statistical re-analysis. The data used for our meta-analysis were primarily extracted from unadjusted results (means, standard deviations for continuous outcomes; *n*, percentage for binary outcomes), which in few instances resulted in marginally different measures of associations compared to adjusted models reported in the original trial publications. However, restriction of our searches to RCTs should reduce concerns of confounding and selection bias and thus these differences in outcome-specific inferences are not expected to result in substantial bias in our meta-analyses. Third, we did not specify sub-group analyses *a priori* as we were not sure which different intervention types had been studied and our review protocol was not pre-registered. While we aimed to report on the complete set of studies, outcomes and interventions that met our eligibility criteria, it is possible that not having published the study protocol prior to conducting the review may have introduced meta-bias. It is also possible that due to publication bias, our review does not reflect a fully representative synthesis of the evidence on the effect of MMH interventions on child development outcomes (Bender *et al*., 2018). To mitigate this potential for publication bias, we searched eight non-academic databases to include unpublished literature meeting our eligibility criteria.

Notwithstanding these limitations, results of this systematic review and meta-analysis are promising and have implications for policy and practice. We identified a sizeable number of RCTs that evaluated the impact of MMH interventions on child-related outcomes in LMICs. Whereas impacts of these interventions on most child outcomes were uncertain, we identified a promising sizeable impact of MMH interventions on rates of exclusive breastfeeding, an outcome of vital public health importance globally. Evidence from this review further supports the importance of improving MMH, which has similarly been recommended by the WHO, as a strategy to further the critical effort to improve child health in LMICs.

## Data Availability

Data extracted from included studies for the narrative review and meta-analysis are available online: https://osf.io/qwdet/.

## Supplementary material

For supplementary material accompanying this paper visit https://doi.org/10.1017/S2045796020000864. click here to view supplementary material
