# Supplementary material for: Impact of maternal mental health interventions on child-related outcomes in low- and middle-income countries: a systematic review and meta-analysis
Source: Epidemiol Psychiatr Sci. 2020 Oct 19;29:e174. doi: 10.1017/S2045796020000864 (PMC7681164; doi:10.1017/S2045796020000864)
Supplement: Supplementary file 1 [file S2045796020000864sup001.zip › S2045796020000864sup001.docx]

**Author(s)**: MP

**Date**: 23 July 2020

**Question**: Maternal mental health interventions compared to standard care for mothers living in low- and middle-income countries

**Setting**: Low- and middle-income countries

| **Certainty assessment** | | | | | | | | | **№ of patients** | | | | **Effect** | | | | **Certainty** | | | **Importance** | |  |
| --- | --- | --- | --- | --- | --- | --- | --- | --- | --- | --- | --- | --- | --- | --- | --- | --- | --- | --- | --- | --- | --- | --- |
| **№ of studies** | | **Study design** | | **Risk of bias** | **Inconsistency** | **Indirectness** | **Imprecision** | **Other considerations** | **Maternal mental health interventions** | | **standard care** | | **Relative (95% CI)** | | **Absolute (95% CI)** | |  |  |  |  |  |  |
| **Exclusive breastfeeding** | | | | | | | | | | | | | | | | | | | | | |  |
| 10 | | randomised trials | | not serious | serious ^a^ | not serious | not serious | none | 470/2505 (18.8%) | | 319/2244 (14.2%) | | **RR 1.39** (1.13 to 1.71) | | **55 more per 1.000** (from 18 more to 101 more) | | ⨁⨁⨁◯ MODERATE | | | CRITICAL | |  |
| **Cognitive development** | | | | | | | | | | | | | | | | | | | | | |  |
| 3 | | randomised trials | | not serious | not serious | not serious | not serious | none | 647 | | 609 | | - | | SMD **0.07 SD higher** (0.04 lower to 0.18 higher) | | ⨁⨁⨁⨁ HIGH | | | CRITICAL | |  |
| **Psychomotor development** | | | | | | | | | | | | | | | | | | | | | |  |
| 2 | | randomised trials | | not serious | not serious | not serious | not serious | none | 276 | | 220 | | - | | SMD **0.05 SD higher** (0.13 lower to 0.23 higher) | | ⨁⨁⨁⨁ HIGH | | | CRITICAL | |  |
| **Not Underweight** | | | | | | | | | | | | | | | | | | | | | |  |
| 4 | | randomised trials | | serious ^b^ | not serious | not serious | not serious | none | 1181/1256 (94.0%) | | 1164/1249 (93.2%) | | **RR 1.00** (0.99 to 1.02) | | **0 fewer per 1.000** (from 9 fewer to 19 more) | | ⨁⨁⨁◯ MODERATE | | | CRITICAL | |  |
| **Weight-for-height** | | | | | | | | | | | | | | | | | | | | | |  |
| 2 | | randomised trials | | serious ^b^ | very serious ^d^ | not serious | not serious | none | 576/615 (93.7%) | | 518/536 (96.6%) | | **RR 0.92** (0.77 to 1.10) | | **77 fewer per 1.000** (from 222 fewer to 97 more) | | ⨁◯◯◯ VERY LOW | | | CRITICAL | |  |
| **Not Stunted** | | | | | | | | | | | | | | | | | | | | | |  |
| 3 | | randomised trials | | serious ^b^ | serious ^e^ | not serious | not serious | none | 885/983 (90.0%) | | 786/897 (87.6%) | | **RR 1.02** (0.97 to 1.08) | | **18 more per 1.000** (from 26 fewer to 70 more) | | ⨁⨁◯◯ LOW | | | CRITICAL | |  |
| **Height** | | | | | | | | | | | | | | | | | | | | | |  |
| 3 | | randomised trials | | serious ^b^ | not serious | not serious | not serious | none | 1189 | | 1138 | | - | | | SMD **0.13 SD higher** (0.02 higher to 0.24 higher) | | ⨁⨁⨁◯ MODERATE | | | CRITICAL |  |
| **Weight** | | | | | | | | | | | | | | | | | | | | | | |
| 5 | | randomised trials | | serious ^b^ | | serious ^f^ | not serious | not serious | none | | 1344 | | 1302 | | - | | SMD **0.16 SD higher** (0.05 lower to 0.36 higher) | | | ⨁⨁◯◯ LOW | | CRITICAL |

**CI:** Confidence interval; **SMD:** Standardised mean difference

#### Explanations

a. I-squared of 61%

b. No information on blinding of outcome assessment and on drop-out rates provided (or high drop-out rates)

c. I-squared of 66%

d. I-squared of 81%

e. I-squared of 57%

f. I-squared of 74%
