# Supplementary figures and images for: Impact of maternal mental health interventions on child-related outcomes in low- and middle-income countries: a systematic review and meta-analysis
Source: Epidemiol Psychiatr Sci. 2020 Oct 19;29:e174. doi: 10.1017/S2045796020000864 (PMC7681164; doi:10.1017/S2045796020000864)

*Supplemental file 2 Meta-analysis of low birth weight*

*
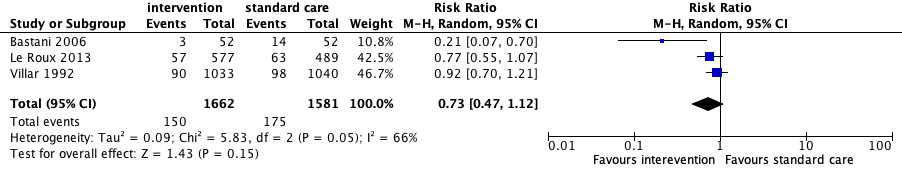
*

Supplement: Supplementary file 1 [file S2045796020000864sup001.zip › S2045796020000864sup002.docx]

*Supplemental file 3 Meta-analysis height*


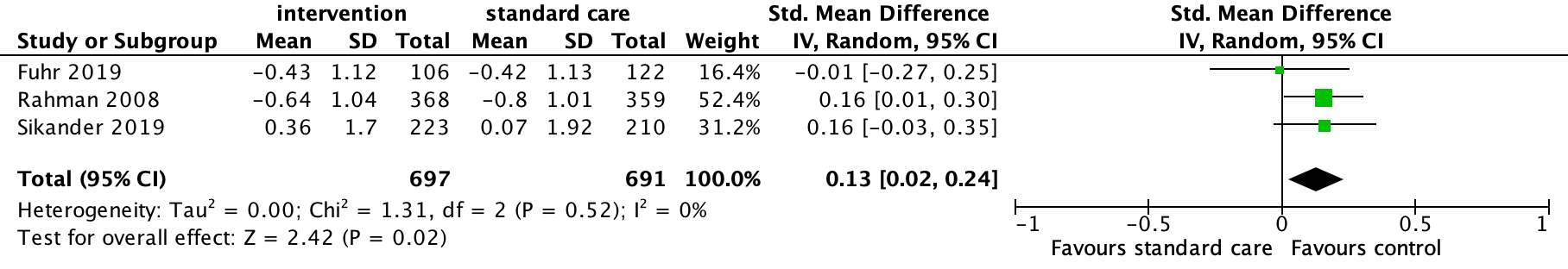

Supplement: Supplementary file 1 [file S2045796020000864sup001.zip › S2045796020000864sup003.docx]

*Supplemental file 4 Meta-analysis weight*


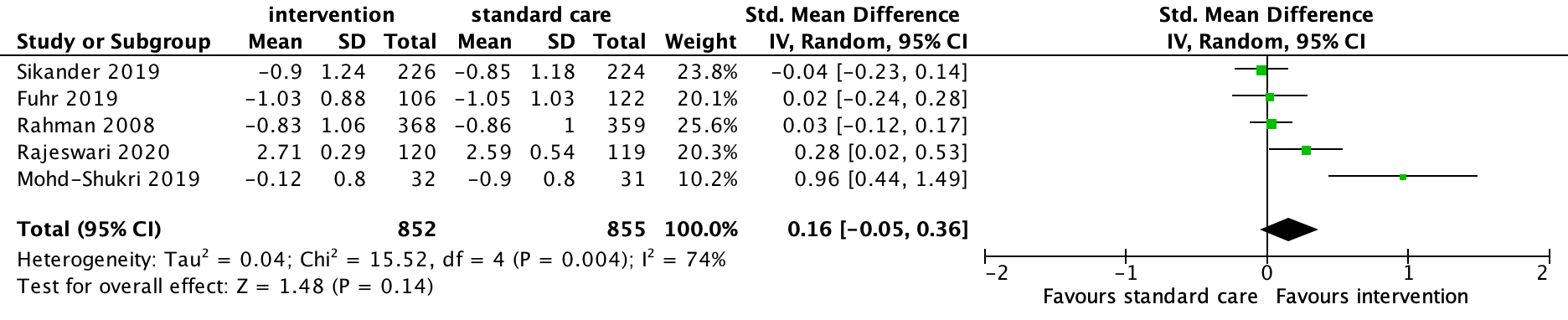

Supplement: Supplementary file 1 [file S2045796020000864sup001.zip › S2045796020000864sup004.docx]

*Supplemental file 5 Meta-analysis weight-for-age (i.e., not underweight)*

*
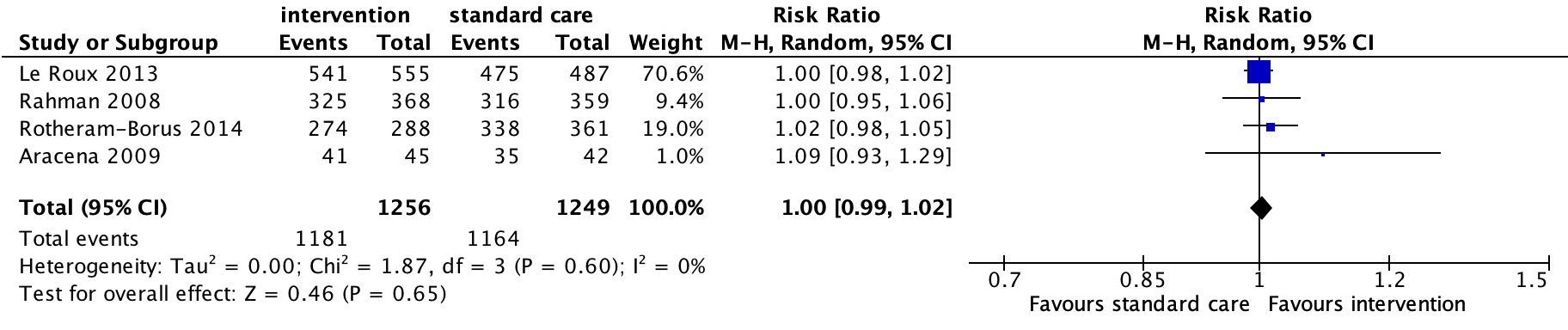
*

Supplement: Supplementary file 1 [file S2045796020000864sup001.zip › S2045796020000864sup005.docx]

*Supplemental file 6 Meta-analysis height-for-age (i.e., not stunted)*

*
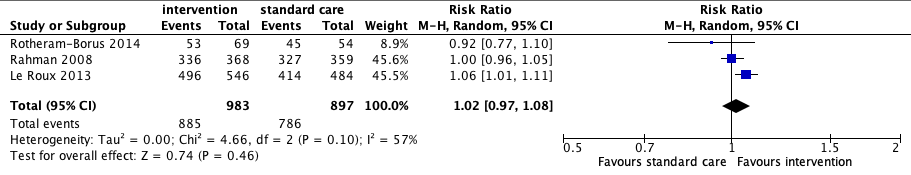
*

Supplement: Supplementary file 1 [file S2045796020000864sup001.zip › S2045796020000864sup006.docx]

*Supplemental file 5 Meta-analysis weight-for-height*

*
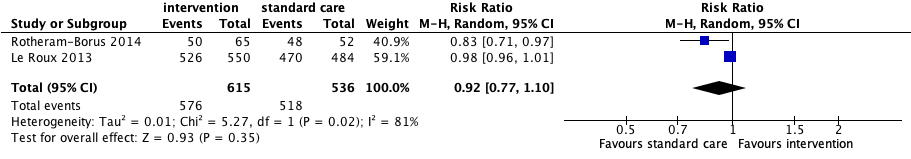
*

Supplement: Supplementary file 1 [file S2045796020000864sup001.zip › S2045796020000864sup007.docx]

*Supplemental file 7 Meta-analysis cognitive development*

*
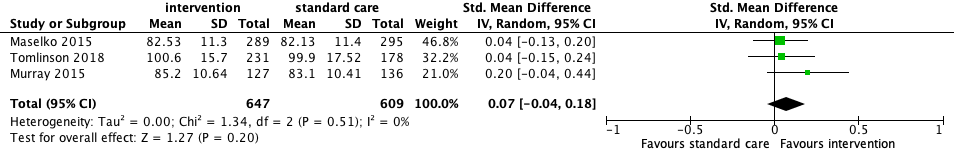
*

Supplement: Supplementary file 1 [file S2045796020000864sup001.zip › S2045796020000864sup008.docx]

*Supplemental file 8 Meta-analysis psychomotor development*


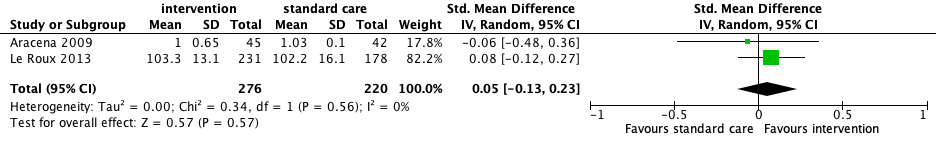

Supplement: Supplementary file 1 [file S2045796020000864sup001.zip › S2045796020000864sup009.docx]
